# A new experimental phase diagram investigation of Cu–Sb

**DOI:** 10.1007/s00706-012-0737-1

**Published:** 2012-08-25

**Authors:** Siegfried Fürtauer, Hans Flandorfer

**Affiliations:** Department of Inorganic Chemistry/Materials Chemistry, University of Vienna, Währingerstraße 42, 1090 Vienna, Austria

**Keywords:** Phase diagrams, Phase transitions, X-ray structure determination, Thermodynamics

## Abstract

**Abstract:**

The binary system Cu–Sb is a constituent system that is studied in investigations of technically important ternary and quaternary alloy systems (e.g., casting alloys and lead-free solders). Although this binary system has been thoroughly investigated over the last century, there are still some uncertainties regarding its high-temperature phases. Thus, parts of its phase diagram have been drawn with dashed lines in reviews published in the literature. The aim of this work was to resolve these uncertainties in the current phase diagram of Cu–Sb by performing XRD, SEM-EDX, EPMA, and DTA. The results from thermal analysis agreed well with those given in the literature, although some modifications due to the invariant reaction temperatures were necessary. In particular, reactions located on the Cu-rich side of the nonquenchable high-temperature β phase (BiF_3_-type) left considerable scope for interpretation. Generally, the structural descriptions of the various binary phases given in the literature were verified. The range of homogeneity of the ε phase (Cu_3_Ti type) was found to be higher on the Sb-rich side. Most of the reaction temperatures were verified, but a few had to be revised, such as the eutectoid reaction $\beta \; \to \;\varepsilon  \; + \;\eta$ at 440 °C (found to occur at 427 °C in this work) and the eutectoid reaction $\gamma  \; \to \;\left( {\text{Cu}} \right) + \delta$ at 400 °C (found to occur at 440 °C in this work). Further phase transformations that had previously only been estimated were confirmed, and their characteristic temperatures were determined.

**Graphical Abstract:**

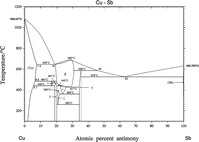

## Introduction

### Lead-free soldering

Compared to, say, lead–acid accumulators, solders used in electronics utilize only a relatively small proportion of the lead consumed worldwide. However, recycling lead from electronic waste is a complicated task, and it pollutes the environment when deposited in landfills and incinerator plants. In the European Union, the use of lead-containing solders has been prohibited since 2006, although there are unfortunately many exceptions for special applications. The electronics industry has therefore tried to phase-in the use of solders containing other, less harmful, materials than lead over the last decade. While the development of lead-free low-temperature soft solders (melting point ~180–230 °C) is fairly advanced, research into lead-free high-temperature soft solders (melting range >230–350 °C) is still in progress. In order to perform a systematic search for appropriate alloy systems, some fundamental data on phase relations and thermochemical properties are essential. COST Action MP0602 will lead to the creation of an encyclopedic database containing data on several different binary and ternary alloy systems. Alloy systems containing the components of lead-free solder and substrate materials are of particular interest for inclusion in this database. The Cu–Sb system is a possible binary constituent of lead-free solder systems. Indeed, Sb is a component of some lead-free solders that are already available on the market (e.g., Ag–Sb–Sn or Cu–Sb–Sn), and copper is the most commonly used substrate, as well as a potential component of the solder itself. 

Despite the fact that there is already a considerable amount of data on the Cu–Sb system, some ambiguities were noticed when a literature search focusing on this system was performed. This primarily affects the hightemperature phase (β phase, BiF_3_ type), which cannot be stabilized at room temperature by quenching. Thus, the aim of the work described in the present paper was to improve the current version of the phase diagram for the Cu–Sb system by incorporating data gained from new experiments and by critically assessing the available data in the relevant literature. This work will therefore contribute valuable information to the lead-free solder database and lead to better thermodynamic descriptions of this binary system (see [1, 2]) and derived higher-order systems via the CALPHAD approach.

### Literature review

**Fig. 1:**
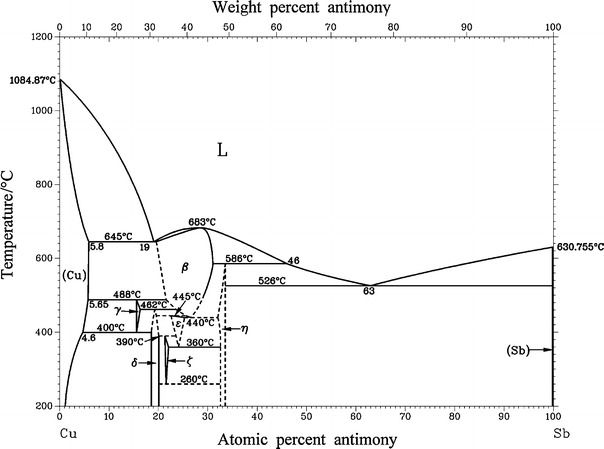
Current version of the phase diagram of the Cu–Sb system [3]

The Cu–Sb phase diagram, as drawn in Massalski [3], is presented in Fig. 1. Invariant reactions are listed in Table 1 and crystallographic data in Table 2, which were taken from works by several authors (see [4–11]).

**Table 1:** Temperature-invariant reactions in the Cu–Sb system [1]

Reaction	Composition /at% Sb			Temp. /°C	Reaction type
${\text{L}} \to \beta$		29		683	Congruent melt
${\text{L}} \to \left( {\text{Cu}} \right) + \beta$	5.8	19	19.5	645	Eutectic
${\text{L}} + \beta \to \eta$	31	33.5	46	586	Peritectic
${\text{L}} \to \eta + \left( {\text{Sb}} \right)$	33.5	63	99.9	526	Eutectic
$\left( {\text{Cu}} \right) + \beta \to \gamma$	5.65	15.5	21.5	488	Peritectoid
$\beta + \gamma \to \delta$	16.5	19	24	462	Peritectoid
$\beta + \delta \to \varepsilon$	19.5	23	25.5	445^a^	Peritectoid
$\beta \to \varepsilon + \eta$	25.5	26.5	32	440^a^	Eutectoid
$\gamma \to \left( {\text{Cu}} \right) + \delta$	4.6	15.5	18.5	400	Eutectoid
$\delta + \varepsilon \to \zeta$	20	21.5	23.5	390^a^	Peritectoid
$\varepsilon \to \zeta + \eta$	22.5	24	32.5	360	Eutectoid
$\zeta \to \delta + \eta$	20	21.5	32.5	260^a^	Eutectoid

^a ^Uncertain values

**Table 2:** Crystallographic data for Cu–Sb phases

Phase	Stoichiometry	Type	Pearson symbol	Space group	No.	*a* /Å	*b* /Å	*c* /Å	Ref.
α	(Cu)	Cu	cF4	*Fm-3m*	225	3.6130	–	–	[4]
β	Cu_3_Sb	BiF_3_	cF16	*Fm-3m*	225	6.0000	–	–	[5]
γ	Cu_4_Sb	Mg	hP2	*P63/mmc*	194	2.7520	–	4.3200	[6]
δ	Cu_78_Sb_20_	Cu_78_Sb_21_	hP98	*P63/mmc*	194	19.124	–	4.324756	[7]
ε	Cu_3_Sb	Cu_3_Ti	oP8	*Pmmn*	59	5.5040	4.3530	4.7680	[8]
ζ	Cu_10_Sb_3_	Cu_10_Sb_3_	hP26	*P-3*	147	9.9200	–	4.3200	[9]
η	Cu_2_Sb	Cu_2_Sb	tP6	*P4/nmm*	129	4.0014	–	6.1044	[10]
θ	(Sb)	As	hR2	*R-3* *m*	166	4.3084	–	11.2740	[11]

The α phase is Cu containing Sb with extended solubility. The maximum solubility of Sb occurs at 5.8 at% Sb and 645 °C. In contrast to this, there is nearly no solubility of Cu in Sb. The β phase, which is a high-temperature phase, melts congruently at 683 °C. It crystallizes in a cubic BiF_3_-type structure (DO_3_) with the space group *Fm-3m*. At the liquid melt, the Sb-rich β forms the η phase in a peritectic reaction (586 °C). On the Cu-rich side, β and (Cu) are formed eutectically at 645 °C. The β phase decomposes in a eutectoid reaction at 440 °C into ε and η. Schubert and Illschner first published this reaction [12], and Heumann and Heinemann [13] subsequently proposed the eutectoid reaction $\beta \to \delta + \varepsilon$ at 436 °C and 22.3 at% Sb based on micrographic data. However, Hansen [14] and later Massalski [3] did not consider the work of Heumann and Heinemann in their assessments, instead establishing the eutectoid decomposition $\varepsilon \to \delta + \eta$ at 375 °C, which was also determined by micrographic data from Heumann and Heinemann’s work [13]. Later, Günzel and Schubert [15] described a new phase (ζ) occurring on the Sb-rich side of δ. Therefore, the latter reaction had to be corrected to $\varepsilon \to \zeta + \eta$ (adjusted from 375 to 360 °C). The γ phase is formed from the β phase with (Cu) in the peritectoid reaction $\left( {\text{Cu}} \right) + \beta \to \gamma$ (488 °C). This transformation and the peritectoid reaction $\gamma + \beta \to \delta$ (462 °C) were found by Murakami and Shibata [16], and both were confirmed by Schubert and Ilschner [12] using dilatometric methods. The ε phase was first mentioned by the same authors. They tentatively fixed the respective reaction temperatures and the concentration limits according to their high-temperature X-ray diffraction results. The invariant peritectoid temperatures of the reactions $\beta + \delta \to \varepsilon$ (445 °C) and $\delta + \varepsilon \to \zeta$(390 °C) as well as the eutectoid decomposition of ζ ($\zeta \to \delta + \eta$, 280 °C) were only roughly estimated by Günzel and Schubert [15] from X-ray diffraction experiments. They proposed the peritectoid temperature ($\delta + \varepsilon \to \zeta$) to be 390 °C, but due to the scatter in their experimental data it can only be said to occur in the temperature range 375–400 °C. The decomposition temperature ($\zeta \to \delta + \eta$) is suggested to be 260 °C, but again this temperature can only be stated to lie between 250 and 300 °C. The experimental evidence for the eutectoid reaction $\gamma \to \left( {\text{Cu}} \right) + \delta$ at 400 °C as presented in the phase diagrams of Hansen [14] and Massalski [3] is unknown. Thus, some reaction temperatures and phase homogeneity ranges are tentative and not determined precisely yet. This is shown by dashed lines in the assessment of the Cu–Sb system by Massalski [3]. Further works by Liu et al. [2] in 2000 and Gierlotka et al. [1] in 2009 contribute thermodynamic assessments with similar transition temperatures to those described by Massalski [3]. These works are the most recent ones; nevertheless, information on the ranges of homogeneity of many phases is missing. Liu et al. [2] modeled the liquid, the (Cu), the (Sb), and the β phases as solid solutions, as did Gierlotka et al. [1], but the latter also calculated the δ and the γ phases as sublattice models. The results obtained in the present work are compared with those given in [3].

## Results and discussion

**Fig. 2:**
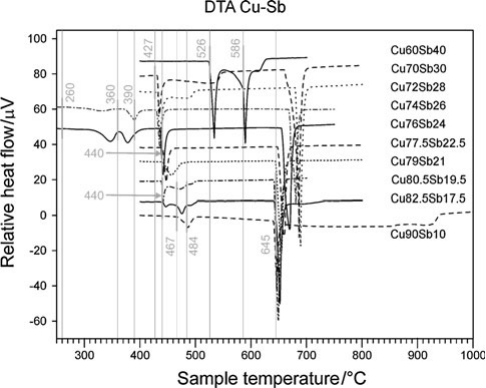
DTA curves of samples with 10–40 at% Sb

**Fig. 3:**
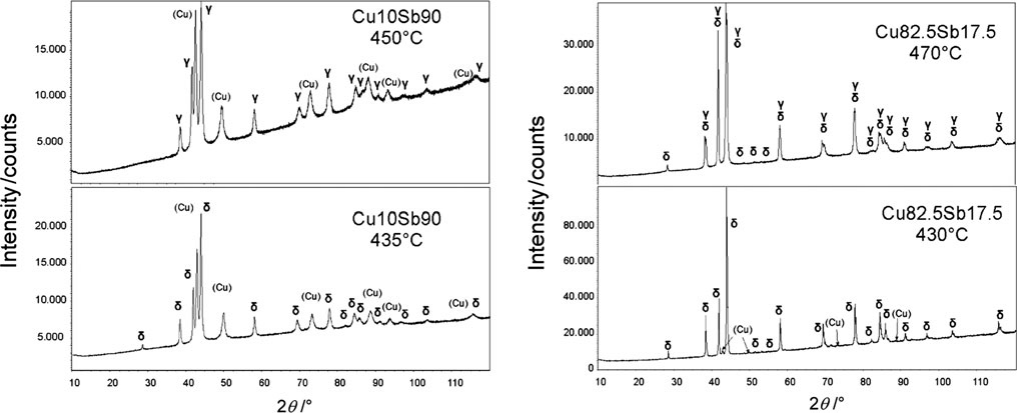
XRD patterns of quenched Cu_90_Sb_10_ and Cu_82.5_Sb_17.5_ samples annealed at different temperatures

The samples used for DTA measurements were annealed for four weeks at 340 °C or six months at 170 °C and quenched in cold water. The temperature program included two heating and cooling loops, starting from the annealing temperature and ending 50–100 °C above the estimated liquidus temperature. The heating rate was 5 K/min, the measured temperatures are summarized in Table 3, the DTA curves can be found in Fig. 2, and the corresponding invariant reactions are listed in Table 4. In addition, we generally performed measurements with heating rates of 10 K/min in order to observe the influence of the heating rate on the characteristic temperatures. There was no significant change in the transition temperatures when the heating rate was increased. The temperatures of the maxima of the melting peaks of all samples are consistent with the liquidus temperatures given in [3]. The solidus of the β phase, which was established by performing DTA measurements of five samples with 21–28 at% Sb, was also in agreement with the literature [3]. The reaction temperature as well as the liquidus concentration of 19 at% Sb for the eutectic reaction located at 645 °C (${\text{L}} \to \left( {\text{Cu}} \right) + \beta$) were confirmed based on three of our samples; see Table 3. However, samples at 10, 17.5, and 19.5 at% Sb showed some discrepancies from the data in the literature at temperatures below 645 °C [3]. Strong effects were observed in all three samples at temperatures of 467 and 484 °C. We allocated the effect at 467 °C to the reaction $\beta + \gamma \to \delta$, which is described in the literature as occurring at 462 °C [3], and the effect at 484 °C to $\left( {\text{Cu}} \right) + \beta \to \gamma$ (which takes place at 488 °C according to the literature [3]). However, according to the phase relations [3], the effect at 467 °C should not be observable in the sample with 10 at% Sb in the first heating run. Surprisingly, this effect was even stronger in the second heating run. In order to clarify this discrepancy, we annealed this sample at 470 and 480 °C for 28 days. Both temperatures resulted in large amounts of (Cu) and γ, but also traces of the β phase (see Tables 5, 6). It is worth noting at this point that the β phase cannot be quenched; it mainly decomposes to the low-temperature phases δ and ε. Thus, we instead assume that (Cu) is in equilibrium with γ at both temperatures. Although the effect is clearly present at 467 °C in the sample with 10 at% Sb, we have decided not to change the previously accepted phase diagram given in the literature [3]. XRD analysis of Cu_90_Sb_10_ annealed at 435 °C and Cu_82.5_Sb_17.5_ annealed at 430 °C showed (Cu) and δ as equilibrium phases (see Fig. 3). According to the literature, these samples should both contain the γ phase [3]. Supported by an invariant reaction observed at 440 °C during DTA of Cu_82.5_Sb_17.5_, we fixed the eutectoid reaction $\gamma \to \left( {\text{Cu}} \right) + \delta$ at this temperature. This is additionally supported by the fact that the original source of the reaction temperature of 400 °C given in [3] could not be found and thus appears to be estimated. The peritectoid reaction $\beta + \delta \to \varepsilon$ was corroborated by DTA of samples with 21 and 22.5 at% Sb. However, the corresponding temperature (440 °C) differs slightly from the literature value (445 °C [3]). DTA of these samples should also show invariant reactions according to $\varepsilon + \delta \to \zeta$ (390 °C) and $\varepsilon \to \zeta + \eta$ (360 °C), and we did indeed find the reaction at 360 °C in Cu_87.5_Sb_22.5_ as a weak effect in the second heating run. However, we could not locate the peritectoid reaction at 390 °C. Thermal analysis of the samples with 24 and 26 at% Sb agreed well with the previously reported phase diagram [3] above 350 °C. On the other hand, DTA of samples annealed at 170 °C did not indicate the invariant reaction at 260 °C $\zeta \to \delta + \eta$. Instead, we found two further signals at different temperatures that are possibly related to this reaction (24 at% Sb 323 °C, 26 at% Sb 302 °C; see also Table 3). Because XRD data for the samples with 21, 22.5, 24, and 26 at% Sb are consistent with the literature data [3], we kept the previously reported phase relations and reaction temperatures. Using the samples with 28 and 30 at% Sb, we were able to determine the temperature of the eutectoid reaction $\beta \to \varepsilon + \eta$ as 427 °C, which had previously been estimated as 440 °C ([3]: dashed lines, see Fig. 1). The liquidus and solidus curves allowed us to estimate the congruent melting point of the β phase at 690 °C and 29 at% Sb ([3], 683 °C). Finally, we also verified the eutectic reaction at 526 °C (${\text{L}} \to \eta + \left( {\text{Sb}} \right)$) and the peritectic reaction at 586 °C ($\beta + {\text{L}} \to \eta$).

**Fig. 4:**
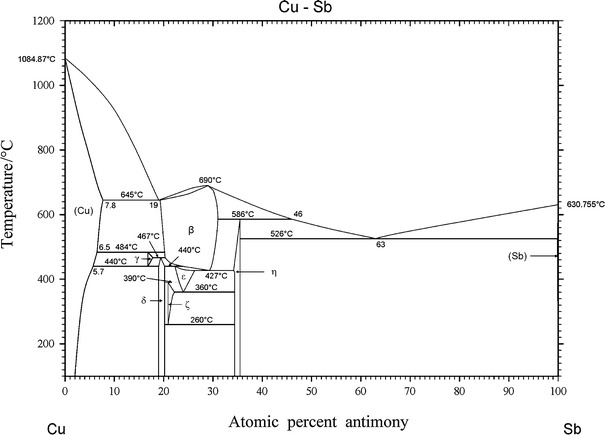
New version of the Cu–Sb phase diagram

To investigate the solubility ranges of the phases, we performed SEM/EDX measurements on polished samples. We were especially interested in determining the ranges of homogeneity of the phases that had been only tentatively fixed in the literature ([3], dashed lines). All of the results of the EDX measurements along with BSE images of the examined samples can be found in Table 7. Overall, the ranges of homogeneity were found to fit well to the currently accepted phase diagram in the literature [3]. The solubility limits indicated by the dashed lines for the ε phase, η phase, and the high-temperature region of the δ phase were determined. For the ε phase, an extension of the phase field to higher Sb concentrations than those estimated in the literature [3] was observed, and the η phase was also found to occur at higher Sb concentrations (see Tables 7, 8; Fig. 4). Even the very narrow two-phase field between the δ and the ζ phases was confirmed by EDX and XRD measurements of the sample with 20.5 at% Sb (see Table 5).

**Table 3:** Summary of measured thermal effects

No.	Nominal comp. /at%	Heat treatment	Thermal analysis			
			Heating /°C			Cooling /°C
			Invariant effects	Other effects	Liquidus	Liquidus
1	Cu_90_Sb_10_	340 °C, 28 days	469, 482.2, 642.2		926.1	920.6
2	Cu_82.5_Sb_17.5_	340 °C, 28 days	440, 467.1, 485, 644.9		651.5	642.2
3	Cu_80.5_Sb_19.5_	340 °C, 28 days	461.8, 486.8	641.8	650.9	641.4
4	Cu_79_Sb_21_	340 °C, 28 days	443.5, 451.9	644.2	654.6	647.5
5	Cu_77.5_Sb_22.5_	340 °C, 28 days	360.5, 441.4	648.9	660.8	655.2
6	Cu_76_Sb_24_	170 °C, 6 months	(323.3)^a^, 363.3	436.8, 655	670.6	665.9
7	Cu_74_Sb_26_	170 °C, 6 months	(302.3)^a^, 375.8, 431.9	668.9	681.6	675.0
8	Cu_72_Sb_28_	340 °C, 28 days	428.6	433.8, 679.7	690.2	679.3
9	Cu_70_Sb_30_	Melt	426.7	470.3, 673.1	686.3	676.7
10	Cu_60_Sb_40_	340 °C, 28 days	525.8, 586.2		616.0	597.3
11	Cu_35_Sb_65_	340 °C, 28 days	524.8		539.9	506.6
12	Cu_30_Sb_70_	340 °C, 28 days	525.4		546.4	518.2
13	Cu_25_Sb_75_	340 °C, 28 days	526.5		553.9	517.7

^a ^Very weak effect

**Table 4:** Comparison of reactions and temperatures published in the literature and found in this work

Invariant reactions	Temp. /°C: [1]	Temp. /°C: this work	Comments
${\text{L}} \to \beta$	683	690	Estimated from liquidus curves
${\text{L}} \to \left( {\text{Cu}} \right) + \beta$	645	645	
${\text{L}} + \beta \to \eta$	586	586	
${\text{L}} \to \eta + \left( {\text{Sb}} \right)$	526	526	
$\left( {\text{Cu}} \right) + \beta \to \gamma$	488	484	
$\beta + \gamma \to \delta$	462	467	
$\beta + \delta \to \varepsilon$	445	440	
$\beta \to \varepsilon + \eta$	440	427	
$\gamma \to \left( {\text{Cu}} \right) + \delta$	400	440	
$\delta + \varepsilon \to \zeta$	390	390	Not detected, adopted from literature
$\varepsilon \to \zeta + \eta$	360	360	
$\zeta \to \delta + \eta$	260	260	Not detected, adopted from literature

**Table 5:** Crystal structures and lattice parameters of quenched Cu–Sb samples

Sample	Heat treatment	Phase	Structure type	Lattice parameter /Å	Comment
Cu_90_Sb_10_	Melt 1,000 °C, 1 day	α = (Cu)	*Fm-3m*	*a* = 3.662(4)	Nonequilibrium (quenched from liquid)
		δ = Cu_78_Sb_20_	*P6*_*3*_ */mmc*	*a* = 19.035(3), *c* = 4.3285(9)	
	340 °C, 28 days	α = (Cu)	*Fm-3m*	*a* = 3.64479(2)	
		δ = Cu_78_Sb_20_	*P6*_*3*_ */mmc*	*a* = 19.0823(2), *c* = 4.32615(9)	
	390 °C, 28 days	α = (Cu)	*Fm-3m*	*a* = 3.65393(6)	
		δ = Cu_78_Sb_20_	*P6*_*3*_ */mmc*	*a* = 19.0421(4), *c* = 4.3260(2)	
	420 °C, 28 days	*α* = (Cu)	*Fm-3m*	*a* = 3.66266(7)	
		δ = Cu_78_Sb_20_	*P6*_*3*_ */mmc*	*a* = 19.0286(5), *c* = 4.3291(2)	
	435 °C, 28 days	α = (Cu)	*Fm-3m*	*a* = 3.66487(9)	
		δ = Cu_78_Sb_20_	*P6*_*3*_ */mmc*	*a* = 19.0074(4), *c* = 4.3281(1)	
	450 °C, 28 days	α = (Cu)	*Fm-3m*	*a* = 3.6686(1)	
		γ = Cu_4_Sb	*P6*_*3*_ */mmc*	*a* = 2.68573(9), *c* = 4.3274(3)	
	470 °C, 28 days	α = (Cu)	*Fm-3m*	*a* = 3.62263(2)	β phase partially stabilized
		β = Cu_3_Sb	*Fm-3m*	*a* = 5.8105(1)	
		γ = Cu_4_Sb	*P6*_*3*_ */mmc*	*a* = 3.3521(3), *c* = 2.8928(5)	
	480 °C, 28 days	α = (Cu)	*Fm-3m*	*a* = 3.6719(1)	β phase partially stabilized
		β = Cu_3_Sb	*Fm-3m*	*a* = 5.9162(9)	
		γ = Cu_4_Sb	*P6*_*3*_ */mmc*	*a* = 2.7527(4), *c* = 4.244(2)	
	600 °C, 28 days	α = (Cu)	*Fm-3m*	*a* = 3.67907(7)	
		γ = Cu_4_Sb	*P6*_*3*_ */mmc*	*a* = 2.74168(8), *c* = 4.3304(2)	
		δ = Cu_78_Sb_20_	*P6*_*3*_ */mmc*	*a* = 19.101(3), *c* = 4.3307(8)	
Cu_82.5_Sb_17.5_	Melt 1,000 °C, 1 day	δ = Cu_78_Sb_20_	*P6*_*3*_ */mmc*	*a* = 19.1198(4), *c* = 4.3273(1)	Nonequilibrium (quenched from liquid)
		ε = Cu_3_Sb	*Pmmn*	*a* = 5.5045(3), *b* = 4.3355(2), *c* = 4.7549(4)	
	340 °C, 28 days	α = (Cu)	*Fm-3m*	*a* = 3.6482(9)	
		δ = Cu_78_Sb_20_	*P6*_*3*_ */mmc*	*a* = 19.0836(1), *c* = 4.32763(4)	
	430 °C, 28 days	α = (Cu)	*Fm-3* *m*	*a* = 3.6676(7)	
		δ = Cu_78_Sb_20_	*P6*_*3*_ */mmc*	*a* = 18.9911(2), *c* = 4.32639(8)	
	470 °C, 28 days	γ = Cu_4_Sb	*P6*_*3*_ */mmc*	*a* = 2.69586(9), *c* = 4.3309(3)	
		δ = Cu_78_Sb_20_	*P6*_*3*_ */mmc*	*a* = 19.0113(5), *c* = 4.3278(2)	
	600 °C, 28 days	α = (Cu)	*Fm-3m*	*a* = 3.6805(4)	β phase partially stabilized
		β = Cu_3_Sb	*Fm-3m*	*a* = 5.9239(5)	
		δ = Cu_78_Sb_20_	*P6*_*3*_ */mmc*	*a* = 19.1485(3), *c* = 4.3291(1)	
Cu_80.5_Sb_19.5_	340 °C, 28 days	δ = Cu_78_Sb_20_	*P6*_*3*_ */mmc*	*a* = 19.1178(1), *c* = 4.32590(6)	
Cu_79.5_Sb_20.5_	170 °C, 6 months	δ = Cu_78_Sb_20_	*P6*_*3*_ */mmc*	*a* = 19.1241(5), *c* = 4.3279(1)	Nonequilibrium (not sufficiently annealed)
		ζ = Cu_10_Sb_3_	*P-3*	*a* = 9.9335(1), *c* = 4.3227(1)	
		η = Cu_2_Sb	*P4/nmm*	*a* = 4.0035(7), *c* = 6.087(2)	
	280 °C, 28 days	δ = Cu_78_Sb_20_	*P6*_*3*_ */mmc*	*a* = 19.1476(2), *c* = 4.32586(9)	
		ζ = Cu_10_Sb_3_	*P-3*	*a* = 9.90716(7), *c* = 4.32301(8)	
	350 °C, 28 days	δ = Cu_78_Sb_20_	*P6*_*3*_ */mmc*	*a* = 19.1604(4), *c* = 4.3249(1)	
		ζ = Cu_10_Sb_3_	*P-3*	*a* = 9.89815(8), *c* = 4.32278(6)	
	420 °C, 28 days	δ = Cu_78_Sb_20_	*P6*_*3*_ */mmc*	*a* = 19.1655(1), *c* = 4.32620(3)	
		ε = Cu_3_Sb	*Pmmn*	*a* = 5.493(2), *b* = 4.3468(2), *c* = 4.757(2)	
Cu_79_Sb_21_	Melt 1,000 °C, 1 day	δ = Cu_78_Sb_20_	*P6*_*3*_ */mmc*	*a* = 19.1665(8), *c* = 4.3317(3)	Nonequilibrium (quenched from liquid)
		ε = Cu_3_Sb	*Pmmn*	*a* = 5.4977(3), *b* = 4.3301(1), *c* = 4.7698(2)	
	340 °C, 28 days	ζ = Cu_10_Sb_3_	*P-3*	*a* = 9.90817(7), *c* = 4.32364(5)	
	430 °C, 28 days	δ = Cu_78_Sb_20_	*P6*_*3*_ */mmc*	*a* = 19.1082(2), *c* = 4.32665(9)	
		ε = Cu_3_Sb	*Pmmn*	*a* = 5.4920(2), *b* = 4.34468(8), *c* = 4.7510(2)	
	470 °C, 28 days	β = Cu_3_Sb	*Fm-3m*	*a* = 6.0108(8)	β phase partially stabilized
		δ = Cu_78_Sb_20_	*P6*_*3*_ */mmc*	*a* = 19.1990(2), *c* = 4.33742(8)	
		ε = Cu_3_Sb	*Pmmn*	*a* = 5.443(1), *b* = 4.3296(3), *c* = 4.711(1)	
Cu_77.5_Sb_22.5_	Melt 1,000 °C, 1 day	δ = Cu_78_Sb_20_	*P6*_*3*_ */mmc*	*a* = 19.2303(6), *c* = 4.3342(2)	Nonequilibrium (quenched from liquid)
		ε = Cu_3_Sb	*Pmmn*	*a* = 5.5054(5), *b* = 4.3409(2), *c* = 4.7631(5)	
	340 °C, 28 days	ζ = Cu_10_Sb_3_	*P-3*	*a* = 9.92390(6), *c* = 4.32223(5)	
		η = Cu_2_Sb	*P4/nmm*	*a* = 4.0018(2), *c* = 6.1031(5)	
	430 °C, 28 days	δ = Cu_78_Sb_20_	*P6*_*3*_ */mmc*	*a* = 19.156(1), *c* = 4.3273(4)	
		ε = Cu_3_Sb	*Pmmn*	*a* = 5.49427(9), *b* = 4.34601(5), *c* = 4.75169(9)	
	450 °C, 28 days	δ = Cu_78_Sb_20_	*P6*_*3*_ */mmc*	*a* = 19.022(5), *c* = 4.4541(9)	Nonequilibrium: β phase decomposed during quenching
		ε = Cu_3_Sb	*Pmmn*	*a* = 5.3831(5), *b* = 4.2871(5), *c* = 5.0467(6)	
		η = Cu_2_Sb	*P4/nmm*	*a* = 4.2732(3), *c* = 5.7367(7)	
	470 °C, 28 days	δ = Cu_78_Sb_20_	*P6*_*3*_ */mmc*	*a* = 18.973(4), *c* = 4.4484(8)	Nonequilibrium: β phase decomposed during quenching
		ε = Cu_3_Sb	*Pmmn*	*a* = 5.3763(7), *b* = 4.2818(5), *c* = 5.0382(5)	
		η = Cu_2_Sb	*P4/nmm*	*a* = 4.2675(2), *c* = 5.7335(7)	
	600 °C, 28 days	δ = Cu_78_Sb_20_	*P6*_*3*_ */mmc*	*a* = 18.992(4), *c* = 4.4519(8)	Nonequilibrium: β phase decomposed during quenching
		ε = Cu_3_Sb	*Pmmn*	*a* = 5.3810(9), *b* = 4.2812(6), *c* = 5.0447(6)	
		η = Cu_2_Sb	*P4/nmm*	*a* = 4.2667(2), *c* = 5.7424(7)	
Cu_76_Sb_24_	170 °C, 6 months	δ = Cu_78_Sb_20_	*P6*_*3*_ */mmc*	*a* = 19.1412(1), *c* = 4.32539(6)	
		η = Cu_2_Sb	*P4/nmm*	*a* = 4.00170(4), *c* = 6.1027(1)	
	280 °C, 28 days	ζ = Cu_10_Sb_3_	*P-3*	*a* = 9.92096(5), *c* = 4.32247(4)	
		η = Cu_2_Sb	*P4/nmm*	*a* = 4.00200(4), *c* = 6.1038(1)	
	340 °C, 28 days	ζ = Cu_10_Sb_3_	*P-3*	*a* = 9.92319(6), *c* = 4.32294(4)	
		η = Cu_2_Sb	*P4/nmm*	*a* = 4.00364(5), *c* = 6.1042(2)	
	400 °C, 28 days	ε = Cu_3_Sb	*Pmmn*	*a* = 5.5064(3), *b* = 4.35302(4), *c* = 4.7680(2)	
Cu_74_Sb_26_	170 °C, 6 months	α = (Cu)	*Fm-3m*	*a* = 3.6216(1)	Nonequilibrium (not sufficiently annealed)
		δ = Cu_78_Sb_20_	*P6*_*3*_ */mmc*	*a* = 19.1335(5), *c* = 4.3248(1)	
		ζ = Cu_10_Sb_3_	*P-3*	*a* = 9.9149(3), *c* = 4.3213(2)	
		η = Cu_2_Sb	*P4/nmm*	*a* = 4.00201(3), *c* = 6.10281(9)	
	280 °C, 28 days	ζ = Cu_10_Sb_3_	*P-3*	*a* = 9.91867(6), *c* = 4.32272(4)	
		η = Cu_2_Sb	*P4/nmm*	*a* = 4.00203(2), *c* = 6.10400(8)	
	340 °C, 28 days	ζ = Cu_10_Sb_3_	*P-3*	*a* = 9.91806(9), *c* = 4.32282(7)	
		η = Cu_2_Sb	*P4/nmm*	*a* = 4.00295(4), *c* = 6.1034(1)	
	400 °C, 28 days	ε = Cu_3_Sb	*Pmmn*	*a* = 5.5090(1), *b* = 4.35420(5), *c* = 4.7757(1)	
		η = Cu_2_Sb	*P4/nmm*	*a* = 4.00147(8), *c* = 6.1038(2)	
Cu_72_Sb_28_	Melt 1,000 °C, 1 day	ε = Cu_3_Sb	*Pmmn*	*a* = 5.5132(2), *b* = 4.35595(8), c = 4.7800(1)	Nonequilibrium (quenched from liquid)
		η = Cu_2_Sb	*P4/nmm*	*a* = 4.00218(5), *c* = 6.1043(1)	
	340 °C, 28 days	ζ = Cu_10_Sb_3_	*P-3*	*a* = 9.9206(1), *c* = 4.3219(1)	
		η = Cu_2_Sb	*P4/nmm*	*a* = 4.00138(3), *c* = 6.1032(1)	
	430 °C, 28 days	ε = Cu_3_Sb	*Pmmn*	*a* = 5.5197(2), *b* = 4.36081(9), *c* = 4.7898(2)	
		η = Cu_2_Sb	*P4/nmm*	*a* = 4.00352(4), *c* = 6.1059(1)	
	600 °C, 28 days	β = Cu_3_Sb	*Fm-3m*	*a* = 5.9979(9)	β phase partially stabilized
		ε = Cu_3_Sb	*Pmmn*	*a* = 5.3919(8), *b* = 4.2688(7), *c* = 5.0588(7)	
		η = Cu_2_Sb	*P4/nmm*	*a* = 4.00016(7), *c* = 6.0977(2)	
Cu_70_Sb_30_	430 °C, 28 days	ε = Cu_3_Sb	*Pmmn*	*a* = 5.5157(2), *b* = 4.35768(8), *c* = 4.7830(1)	
		η = Cu_2_Sb	*P4/nmm*	*a* = 4.00130(3), *c* = 6.10265(8)	
	470 °C, 28 days	β = Cu_3_Sb	*Fm-3m*	*a* = 6.0384(9)	β phase partially stabilized
		ε = Cu_3_Sb	*Pmmn*	*a* = 5.5113(5), *b* = 4.3558(2), *c* = 4.7796(4)	
		η = Cu_2_Sb	*P4/nmm*	*a* = 4.00302(2), *c* = 6.10545(7)	
	600 °C, 28 days	β = Cu_3_Sb	*Fm-3m*	*a* = 6.035(3)	β phase partially stabilized
		ε = Cu_3_Sb	*Pmmn*	*a* = 5.5039(3), *b* = 4.3540(1), *c* = 4.7787(2)	
		η = Cu_2_Sb	*P4/nmm*	*a* = 4.00170(3), *c* = 6.10306(8)	
Cu_60_Sb_40_	Melt 1000 °C, 1 day	η = Cu_2_Sb	*P4/nmm*	*a* = 4.00231(2), *c* = 6.10442(8)	Nonequilibrium (quenched from liquid)
		θ = (Sb)	*R-3m*	*a* = 4.3060(1), *c* = 11.2701(7)	
	340 °C, 28 days	η = Cu_2_Sb	*P4/nmm*	*a* = 4.00172(3), *c* = 6.10443(9)	
		θ = (Sb)	*R-3m*	*a* = 4.3066(1), *c* = 11.2689(6)	
	470 °C, 28 days	η = Cu_2_Sb	*P4/nmm*	*a* = 4.00204(2), *c* = 6.10431(7)	
		θ = (Sb)	*R-3m*	*a* = 4.30686(9), *c* = 11.2708(5)	
	600 °C, 28 days	η = Cu_2_Sb	*P4/nmm*	*a* = 4.00239(2), *c* = 6.10462(8)	Nonequilibrium (quenched from liquid)
		θ = (Sb)	*R-3m*	*a* = 4.3072(1), *c* = 11.2720(5)	
Cu_35_Sb_65_	340 °C, 28 days	η = Cu_2_Sb	*P4/nmm*	*a* = 4.00106(5), *c* = 6.1026(1)	
		θ = (Sb)	*R-3m*	*a* = 4.30569(6), *c* = 11.2658(3)	
	535 °C, 28 days	η = Cu_2_Sb	*P4/nmm*	*a* = 4.00165(5), *c* = 6.1029(1)	Nonequilibrium (quenched from liquid)
		θ = (Sb)	*R-3m*	*a* = 4.30639(4), *c* = 11.2707(2)	
Cu_30_Sb_70_	340 °C, 28 days	η = Cu_2_Sb	*P4/nmm*	*a* = 4.00218(4), *c* = 6.1043(1)	
		θ = (Sb)	*R-3m*	*a* = 4.30709(5), *c* = 11.2701(2)	
	535 °C, 28 days	η = Cu_2_Sb	*P4/nmm*	*a* = 4.00065(7), *c* = 6.1018(2)	Nonequilibrium (quenched from liquid)
		θ = (Sb)	*R-3m*	*a* = 4.30563(4), *c* = 11.2650(2)	
Cu_25_Sb_75_	340 °C, 28 days	η = Cu_2_Sb	*P4/nmm*	*a* = 4.00206(5), *c* = 6.1040(1)	
		θ = (Sb)	*R-3m*	*a* = 4.30703(4), *c* = 11.2702(2)	
	535 °C, 28 days	η = Cu_2_Sb	*P4/nmm*	*a* = 4.00151(7), *c* = 6.1029(2)	Nonequilibrium (quenched from liquid)
		θ = (Sb)	*R-3m*	*a* = 4.30667(7), *c* = 11.2690(2)

**Table 6:** Detected phases in quenched samples

Sample	Annealing temperature (°C)															
	Melt	170	280	340	350	420	390	400	430	420	435	450	470	480	535	600
Cu_90_Sb_10_	**(Cu) δ**			(Cu) δ			(Cu) δ			(Cu) δ	(Cu) δ	(Cu) δ	*(Cu) β, γ*	*(Cu) β, γ*		*(Cu) γ, δ*
Cu_82.5_Sb_17.5_	**δ, ε**			(Cu) δ					Cu, δ				γ, δ			*(Cu) β, δ*
Cu_80.5_Sb_19.5_				δ												
Cu_79.5_Sb_20.5_		**δ, ζ, η**	δ, ζ		δ, ζ	δ, ε										
Cu_79_Sb_21_	**δ, ε**			ζ					δ, ε				*β, δ, ε*			
Cu_77.5_Sb_22.5_	**δ, ε**			ζ, η					δ, ε			*δ, ε, η*	*δ, ε, η*			*δ, ε, η*
Cu_76_Sb_24_		δ, η	ζ, η	ζ, η				ε								
Cu_74_Sb_26_		**(Cu) δ, ζ, η**	ζ, η	ζ, η				ε, η								
Cu_72_Sb_28_	**ε, η**			ζ, η					ε, η							*β, ε, η*
Cu_70_Sb_30_									ε, η				*β, ε, η*			*β, ε, η*
Cu_60_Sb_40_	**η, (Sb)**			η, (Sb)									η, (Sb)			**η, (Sb)**
Cu_35_Sb_65_				η, (Sb)											**η, (Sb)**	
Cu_30_Sb_70_				η, (Sb)											**η, (Sb)**	
Cu_25_Sb_75_				η, (Sb)											**η, (Sb)**	

*Bold-underlined* quenched from liquid, *bold* insufficiently annealed, *italic-underlined* decomposition of β phase

**Table 7:** ESEM/EPMA results of Cu–Sb phase compositions

Sample	Ann. temp. /°C	Phase 1 (dark)			Phase 2 (bright)			SEM image
			at% Cu	at% Sb		at% Cu	at% Sb	
Cu_90_Sb_10_	600	(Cu)	93.0	7.0	β_Cu_	80.2	19.8	
	450	(Cu)	93.9	6.1	γ_Cu_	82.3	17.7	
	340	(Cu)	96.0	4.0	δ_Cu_	78.8	21.2	
Cu_82.5_Sb_17.5_	600	(Cu)	93.7	7.0	δ_Cu_	81.0	19.0	
	470	γ_Sb_	81.7	18.3	δ_Cu_	80.0	20.0	
Cu_79.5_Sb_20.5_	350	ζ_Cu_	77.2	22.8	δ_Sb_	78.0	22.0	
Cu_79_Sb_21_	470	δ_Sb_	79.5	20.5	ε_Cu_	76.2	23.8	
Cu_77.5_Sb_22.5_	430	δ_Sb_	79.6	20.4	ε_Cu_	76.7	23.4	
Cu_76_Sb_24_	170	δ_Sb_ (+ small crystals of η)	77.5	22.5	η_Cu_	66	34.0	
Cu_74_Sb_26_	400	ε_Sb_	74.4	25.6	η_Cu_	65.2	34.8	
Cu_72_Sb_28_	430	ε_Sb_	73.7	26.3	η_Cu_	64.7	35.3	
	340	ζ_Sb_	78.0	22.0	η_Cu_	65.5	34.5	
Cu_60_Sb_40_	470	η_Sb_	64	36	(Sb)	0.1	99.9

**Table 8:** Comparison of temperature-invariant reactions in the Cu–Sb system in this work and in [1]

Reaction	Composition /at% Sb			Temp. /°C	Reaction type
${\text{L}} \to \beta$		29.0 (29.0)		690 (683)	Congruent melt
${\text{L }} \to \left( {\text{Cu}} \right) + \beta$	7.8 (5.8)	17.7 (19)	20.4 (19.5)	645 (645)	Eutectic
${\text{L}} + \beta \to \eta$	31.0^b^	35.5 (33.5)	46.0^b^	586 (586)	Peritectic
${\text{L }} \to \eta + \left( {\text{Sb}} \right)$	35.5 (33.5)	63.0^b^	99.9 (99.9)	526 (526)	Eutectic
$\left( {\text{Cu}} \right) + \beta \to \gamma$	6.4 (5.65)	16.5 (15.5)	20.7 (21.5)	484 (488)	Peritectoid
$\beta + \gamma \to \delta$	17.5 (16.5)	19.5 (19.0)	21.5 (24.0)	467 (462)	Peritectoid
$\beta + \delta \to \varepsilon$	20.2 (19.5)	22.3 (23.0)	23.9 (25.5)	440 (445^a^)	Peritectoid
$\beta \to \;\varepsilon + \eta$	26.3 (25.5)	29.3 (26.5)	34.2 (32.0)	427 (440^a^)	Eutectoid
$\gamma \to \left( {\text{Cu}} \right) + \delta$	5.0 (4.6)	16.5 (15.5)	19.0 (18.5)	440 (400)	Eutectoid
$\delta + \varepsilon \to \zeta$	20.2 (20.0)	20.8 (21.5)	23.2 (23.5)	390^a,b^	Peritectoid
$\varepsilon \to \zeta + \eta$	22.2 (22.5)	24.0 (24.0)	34.4 (32.5)	360 (360)	Eutectoid
$\zeta \to \delta + \eta$	20.2 (20.0)	20.8 (21.5)	34.4 (32.5)	260 (260^a^)	Eutectoid

Values in parentheses are from the literature [1]

^a^ Uncertain values

^b^ Value from [1]

## Experimental

### Sample preparation

Samples with 10–75 at% Sb (see Table 9) were prepared from 99.98% Cu (Goodfellow, Cambridge, UK; treated under an H_2_ flow at 200 °C for 5 h to remove oxide layers) and 99.999% Sb (Alfa Aesar, Karlsruhe, Germany; the surface oxide layer was removed by filtration of the melt through quartz glass wool). Weighed amounts of the metals were sealed in quartz glass ampoules under vacuum (~10^−3^ mbar) and alloyed in a resistance furnace at 1,000 °C for a few hours. Annealing was performed again in evacuated quartz glass ampoules for 28 days at selected temperatures (170–600 °C, annealing time at 170 °C was 6 months). Finally, the alloys were quenched in cold water.

**Table 9:** Annealing temperatures

Sample	Annealing temperature /°C															
Cu_90_Sb_10_	Melt			*340*			**390**		**420**		435	**450**	**470**	**480**		**600**
Cu_82.5_Sb_17.5_	Melt			*340*						**430**			**470**			**600**
Cu_80.5_Sb_19.5_	Melt			*340*												
Cu_79.5_Sb_20.5_	Melt	**170**	**280**		**350**	**420**										
Cu_79_Sb_21_	Melt			*340*						**430**			**470**			
Cu_77.5_Sb_22.5_	Melt			*340*						**430**		**450**	**470**			**600**
Cu_76_Sb_24_	Melt	*170*	**280**	*340*				**400**								
Cu_74_Sb_26_	Melt	*170*	**280**	*340*				**400**								
Cu_72_Sb_28_	Melt			*340*						**430**						**600**
Cu_70_Sb_30_	Melt									**430**			**470**			**600**
Cu_60_Sb_40_	Melt			*340*									**470**			**600**
Cu_35_Sb_65_	Melt			*340*											535	
Cu_30_Sb_70_	Melt			*340*											535	
Cu_25_Sb_75_	Melt			*340*											535	

*Underlined* only XRD, *bold* XRD/ESEM, *italics* XRD/ESEM/DTA

### Analytical methods

Experimental techniques applied were powder X-ray diffraction (XRD), thermal analysis (DTA), and metallographic methods (EPMA/ESEM). Thermal analysis was done with a TG/DTA Setsys Evolution instrument from Setaram. The measurements were performed in open alumina crucibles under an Ar atmosphere; slices of Ti sheet in the second crucible were used as reference material.

The powder XRD measurements were done on a Bruker D8 diffractometer (*θ*/2*θ* geometry) at ambient temperature. X-rays were produced in a copper radiation source at an accelerating voltage of 40 kV and with an electron current of 40 mA. A Ni filter was used to remove the K_β_ radiation. The powder was fixed with petroleum jelly on a silicon monocrystal sample carrier, which was rotated during the measurement. The detection unit was the Lynxeye strip detector. Rietveld refinement of the data was done with the Topas3^®^ software provided by Bruker AXS.

An optical microscope (Zeiss Axiotech 100 reflected light microscope) as well as EDX techniques (energy-dispersive spectroscopy; ESEM Zeiss Supra 55 VP) were used for metallographic investigations. In the ESEM, the excitation energy of the electron beam was 15–20 kV. Backscattered electrons were detected in order to visualize the surfaces of our samples. The characteristic spectral lines were used for EDX: the Cu K line and the Sb L line.
